# Serial Measurements of Apoptotic Cell Numbers Provide Better Acceptance Criterion for PBMC Quality than a Single Measurement Prior to the T Cell Assay

**DOI:** 10.3390/cells4010040

**Published:** 2015-01-09

**Authors:** Marie Wunsch, Richard Caspell, Stefanie Kuerten, Paul V. Lehmann, Srividya Sundararaman

**Affiliations:** 1 Department of Anatomy and Cell Biology, University of Wuerzburg, Koellikerstr. 6, 97070 Wuerzburg, Germany; E-Mails: marie.wunsch@uni-wuerzburg.de (M.W.); stefanie.kuerten@uni-wuerzburg.de (S.K.); 2 Cellular Technology Ltd., 20521 Chagrin Blvd., Shaker Hts., OH 44122, USA; E-Mails: richard.caspell@immunospot.com (R.C.); pvl@immunospot.com (P.V.L.)

**Keywords:** T cell assay, apoptosis, acceptance, viability, ELISPOT

## Abstract

As soon as Peripheral Blood Mononuclear Cells (PBMC) are isolated from whole blood, some cells begin dying. The rate of apoptotic cell death is increased when PBMC are shipped, cryopreserved, or stored under suboptimal conditions. Apoptotic cells secrete cytokines that suppress inflammation while promoting phagocytosis. Increased numbers of apoptotic cells in PBMC may modulate T cell functions in antigen-triggered T cell assays. We assessed the effect of apoptotic bystander cells on a T cell ELISPOT assay by selectively inducing B cell apoptosis using α-CD20 mAbs. The presence of large numbers of apoptotic B cells did not affect T cell functionality. In contrast, when PBMC were stored under unfavorable conditions, leading to damage and apoptosis in the T cells as well as bystander cells, T cell functionality was greatly impaired. We observed that measuring the number of apoptotic cells before plating the PBMC into an ELISPOT assay did not reflect the extent of PBMC injury, but measuring apoptotic cell frequencies at the end of the assay did. Our data suggest that measuring the numbers of apoptotic cells prior to and post T cell assays may provide more stringent PBMC quality acceptance criteria than measurements done only prior to the start of the assay.

## 1. Introduction

T cell monitoring studies, e.g., assessing whether a vaccination has induced immunity, require the use Peripheral Blood Mononuclear Cells (PBMC). PBMC are perishable live cells, some of which start dying immediately after isolation from whole blood. A major challenge in the immune monitoring field has been to establish protocols that define proper methods for isolation, shipping and storage of PBMC until they can be tested without loss or change of the functionality of these cells. Under ideal conditions, the PBMC can be tested immediately following isolation at the site where they were obtained. Many times, however, this is unfeasible for large multi-center clinical studies. It would require that each site that participates in the immune monitoring study has a testing laboratory, ideally one that is Good Laboratory Practices (GLP) certified. It also requires that the blood be processed and the cells tested one sample at a time as the donors/subjects become available, irrespective of the time of the day or week. This is an uneconomical practice since testing a single PBMC sample takes essentially the same amount of time and effort as testing many samples simultaneously. Moreover, since the different PBMC samples are not tested side by side but in different experiments, the data are not fully comparable to one another.

A major step towards moving T cell immune monitoring into the realm of feasibility was when we showed that PBMC can be cryopreserved and stored without loss of function [1]. When frozen according to a modified protocol, the number of spot forming units (SFU) in an antigen-specific ELISPOT assay was the same for freshly isolated and cryopreserved CD4 and CD8 cells secreting IL-2, IL-4, IL-5 and IFN-γ [1]. Since then, many laboratories have successfully used cryopreserved PBMC in ELISPOT and other assays to detect the effect of vaccination with HIV antigens, H3N2 hemagluttinin, tuberculosis antigens, and Type-1 diabetes [2,3,4,5,6,7,8]. The key to successful cryopreservation of PBMC is adding warm cryopreservation medium and keeping the cells warm, ideally at 37 °C all until freezing [1]. Also, for thawing cryopreserved PBMC, best results are obtained when bringing the temperature of the cells rapidly to 37 °C [9]. Contrary to popular belief at the time [10] we identified that human white blood cells do not tolerate exposure to pre-chilled media, neither during freezing nor upon thawing. A likely explanation for this finding is that Dimethyl Sulfoxide (DMSO) is less toxic than initially thought and PBMC can withstand up to 30 min exposure to 10% DMSO at 37 °C without loss of function [9]. In contrast, cells at 4 °C are metabolically inactive and cannot actively compensate for the change in osmotic pressure, as DMSO is added during the freezing process or when the DMSO is washed away upon thawing. Also for shipping blood or handling/storing PBMC the cells will fare much better when kept warm, as compared to when they are chilled. Such advances have resulted in successful standardization of T cell monitoring [11,12,13].

For clinical trials it would be essential to be able to reliably assess whether an individual PBMC sample to be tested has suffered damage during shipment, processing, cryopreservation, and storage. Even when strictly adhering to SOPs, unrecorded or uncontrolled events can critically influence the cells’ viability and functionality. For example, frozen PBMC are highly sensitive to fluctuations of temperature during storage [14]. Since there are many parameters in PBMC processing and storage that can affect the functionality of T cells, efforts have been made to come up with acceptance criteria for processed PBMC. These include assessing the live/dead cell ratio for PBMC, the percentage of apoptotic cells measured prior to testing, non-antigen specific functional responses to Phytohemagglutinin (PHA), and reference antigen-specific functional responses for CD4 and CD8 cells [14].

Viability detection using Trypan Blue has been commonly used to identify the percentage of live cells in a PBMC population. Smith *et al.* have suggested that acceptance criteria for a healthy PBMC sample should have a viability >89% when tested with Trypan Blue [14]. We, and others, have noted that Trypan Blue is not ideal for measurement of cell viability due to staining artifacts [15], large numbers of false positive “dead cells” resulting from cells with a reversible damage of their cell membrane [16], and false negatives from cells that have already initiated the apoptotic pathway but still have intact cell membranes. Alternatively, Acridine Orange and Propidium Iodide staining has been shown to be a more accurate means for detecting live and dead cells, respectively [15].

Several methods are used to identify apoptotic cells. One prevalent method is to detect the flipping of Phosphatidylserine (PS) in the cell membrane by Annexin binding. Since PS flipping is potentially reversible, Annexin staining is not a definite marker for apoptosis [16]. The Yo-Pro family of dyes is also commonly used for detecting apoptotic cells. These are monomeric cyanine dyes that bind to nucleic acids of cells. Since normally, these dyes are impermeable to cell membranes, they bind to DNA in apoptotic cells with compromised cell membranes. The Yo-Pro family of dyes acts in a Calcium-independent, non-reversible manner [17] and therefore is a more accurate marker for apoptosis.

Among the various acceptance criteria for PBMC, measurement of the numbers of apoptotic cells prior to performing a cellular assay has been established as the most accurate. In a landmark publication, the acceptance criteria for PBMC were suggested to be >89% viable cells with the fraction of apoptotic cells not exceeding 18% [14].

In this study, we show that mere measurements of live/dead ratios and apoptotic cell frequencies prior to seeding the PBMC into a T cell assay are not necessarily reliable markers for PBMC functionality. Measuring the apoptotic cell fraction at the beginning and at the end of the assay, however, was found to be a more reliable marker to detect damage to PBMC and therefore their functional impairment.

In this study we also addressed the question of whether the presence of apoptotic bystander cells affects T cell functionality. Apoptotic cells are known to send complex signals to macrophages, entailing “find me”, “eat me”, and “do not eat me” messages that direct the clearance of apoptotic cells while preventing pro-inflammatory reactions by the phogocytosing macrophages. The latter protects healthy bystander cells from being damaged [18]. Some of the relevant signaling molecules are found on the cell surface of apoptotic cells such as Phosphatidylserine [19] or ICAM-3 [20]. A change in cell surface charge is also perceived by macrophages as an indicator of apoptosis [21]. Other signaling molecules are secreted by apoptotic cells acting as chemotractors to macrophages. They include Lysophosphatidylcholine (LPC) [22], Annexin-1 [23], Fractalkine [24], and Lactoferrin [25]. On the other hand macrophages, upon apoptotic cell engulfment, secrete anti-inflammatory cytokines such as TGF-β and IL-10 [26,27]. Since all these processes could potentially affect T cell activation and function, we tested whether the presence of apoptotic bystander cells present PBMC would affect the results of T cell ELISPOT assays.

## 2. Experimental Section

### 2.1. Thawing and Handling of PBMC

Cryopreserved PBMC from healthy human donors were obtained from a library of characterized frozen PBMC (ePBMC, CTL, OH). PBMC cryovials stored in Liq.N_2_ vapor phase were transferred to dry ice in Styrofoam containers for transport to the laboratory. PBMC were thawed following a protocol that we have established to provide the optimal recovery and functionality for cryopreserved PBMC [9]. Briefly, to rapidly warm the cells up to 37 °C, the cryovials were placed in a bead bath (CTL-BB-001, CTL, OH) for 8 min. Cryovials were inverted two times and the cell suspension was gently aspirated using a wide bore pipette for transfer to a 15 mL falcon tube. Cryovials were rinsed with 1 mL CTL Anti-Aggregate Wash™ Medium (CTL-AA-005) which was kept at 37 °C. An additional 8 mL of CTL Anti-Aggregate Wash™ Medium at 37 °C was added to the Falcon tube. Cells were centrifuged at 1200 rpm for 10 min and the supernatant was discarded. Following the wash procedure, PBMC were resuspended at a final concentration of 2.5 × 10^6^ PBMC/mL in CTL-Test Medium (CTLT-005) of which 100 μL (2.5 × 10^5^ cells) was plated per well into the ELISPOT assay.

### 2.2. Antigens

The antigens CEF peptide pool (CTL-CEF-002), CMV pool (PA-CMV-001), Flu pool (PA-Flu-001), and EBV pool (PA-EBV-001) were obtained from Cellular Technology Ltd., OH USA. These antigens were used at a final concentration of 2 µg/mL. Mumps antigen (Lot# IV 0094) was obtained from BioWhittaker, MD USA and Mosquito antigen (Lot # 28758) was from Greer (NC, USA). Mumps antigen was used a dilution of 1:80 and Mosquito antigen was used at 10 µg/mL.

### 2.3. Human Interferon-γ ELISPOT Assay

The human interferon-γ ImmunoSpot® kit (CTL-HIFNG-1/5M) was provided by CTL. The assay was performed according to the manufacturer’s recommendations. The PVDF membrane was coated by adding capture antibody overnight. . Antigens were plated in a final volume of 100 μL per well in the above specified concentrations. The antigens were dissolved in CTL Test Medium (CTLT-005) which also constituted the negative/medium control. The plates containing the antigen were stored at 37 °C in a humidified CO_2_ incubator until the cells were ready for plating. The thawed PBMC were added at 2.5 × 10^5^/well using wide-bore pipette tips. Plates were gently tapped on each side to ensure even distribution of the cells as they settled, and incubated for 24 h at 37 °C in a humidified CO_2_ incubator. Following completion of the ELISPOT detection, the plates were air-dried in a laminar flow hood prior to analysis.

ELISPOT plates were scanned and analyzed using an ImmunoSpot® S6 Ultimate Reader from CTL. Spot Forming Units (SFU) were automatically calculated by the ImmunoSpot® Software for each antigen stimulation condition and the medium (negative) control using the SmartCount™ and Autogate™ functions [28]. In all experiments, the negative control wells had less than 10 SFU per 10^6^ PBMC plated.

### 2.4. Viability and Apoptosis Detection

Fluorescence imaging was used to detect live, dead, and apoptotic cells in the PBMC samples. The CTL Cell Counting Reagent (CTL-LDC-100) was mixed with Po-Pro-1™ (Invitrogen, Cat# P3581). This dye mix was added to cells in an equal ratio. Cells were loaded onto disposable hemocytometers (CTL-LDC-100) and imaged using the CTL S6ULT-9000 Analyzer from CTL using the L/D/A Cell Counting software. Following image acquisition, the live, dead, and apoptotic numbers and their relative percentages were automatically computed.

### 2.5. B cell Separation and Apoptosis Induction

Anti-CD20 mAb (Rituximab) was a kind gift from Dr. Donald Anthony from Case Western Reserve University, Cleveland OH. Dynabeads M-280 Tosylactivated (Invitrogen Cat# 14203/4) was coupled to anti-CD20 mAb as per the manufacturer’s instructions. Briefly, dynabeads and the antibody were resuspended in a 0.1 M Borate buffer. 3 µg of the antibody was bound to 10^7^ Dynabeads. The antibody and Dynabeads were vortexed for 1 min. This was incubated for 16–24 h at 37 °C using slow tilt rotation. After incubation, the tube was placed in the magnet (Invitrogen DynaMag™ 5 Cat# 12303D) for 2 min and unbound antibody was discarded. The antibody coupled beads were washed two times with a 3 M ammonium sulfate buffer for 5 min at 4 °C, once with PBS with 1% *w*/*v* BSA for 24 h at 37 °C, and once more with 3M ammonium sulfate for 5 min at 4 °C.

To isolate B cells from PBMC, 2.5 × 10^7^ cells in 1 mL of isolation buffer (PBS with 0.1% BSA and 2 mM EDTA) and 80 µL of the Dynabead-antibody complex was resuspended and incubated at 4 °C with gentle rotation. The tube was placed in a magnet and the supernatant was transferred to another tube. The beads and cells were washed two times in isolation buffer and the supernatant was transferred following magnetic separation. Cells were resuspended in appropriate media following B cell separation from PBMC.

## 3. Results and Discussion

### 3.1. Detecting Live, Dead and Apoptotic Cells

**Figure 1:**
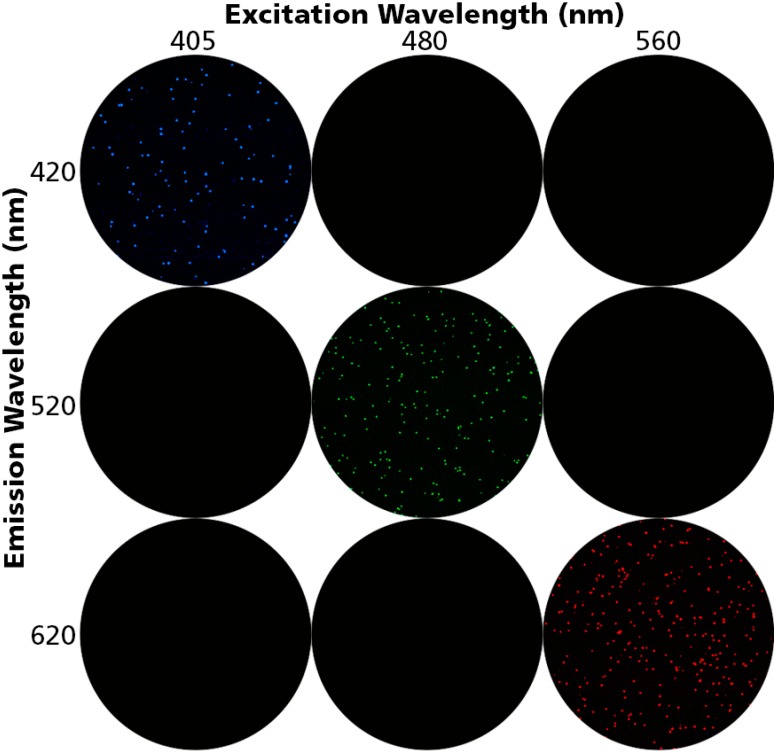
Unambiguous detection of Peripheral Blood Mononuclear Cells (PBMC) positive for Acridine Orange, Propidium Iodide, and Po-Pro-1. PBMC were stained with either Acridine Orange, Propidium Iodide, or Po-Pro-1™ and were imaged at the specified excitation and emission wavelengths. AO-stained (live) cells are detected at 480/520 nm only, PI-stained (dead) cells at (560/620 nm) only, Po-Pro-1 stained (apoptotic) cells at 405/420 nm only. No cross bleeding of signal between the dyes is detectable, providing unambiguous detection of each cell type.

We utilized Acridine Orange (AO), Propidium Iodide (PI), and Po-Pro-1™ to detect live, dead, and apoptotic cells. All three dyes have distinct emission and excitation spectra with little to no overlap between them. Cryopreserved PBMC were thawed, divided into three aliquots, each of which was mixed with each of the three dyes. The cells were interrogated using the excitation and emission filters appropriate for each of the dyes; namely 480 nm and 520 nm for AO, 405 nm and 420 nm for Po-Pro-1™, and 520 nm and 620 nm for PI. As can be seen in Figure 1, each staining was detected in its specific excitation/emission setting only. Therefore, when these settings are used, there is no leaching of signal for the three dyes eliminating false positive staining.

In order to test whether live and dead cells are counted accurately with this dye combination, we stained PBMC obtained within an hour after the blood draw (that should be mostly alive), and the same PBMC after 10 min exposure to 95% Ethanol (that should be mostly dead). All the former stained with AO but not with PI, and all the latter stained with PI and not with AO (data not shown). Live and dead cells were, therefore, were detected by AO/PI staining correctly.

We also validated the counting of live cells in cryopreserved PBMC that have been thawed. Due to the freeze-thaw process in cryopreserved PBMC, live cell numbers, even within different vials of the same donor, vary among each other. The cells were stained with AO/PI, and were counted under the fluorescence microscope and the number of cells appearing green was counted by eye. The same cell samples were counted in an automated fashion on an ImmunoSpot® Ultimate analyzer using the LDA software. The comparisons of machine generated and visual counts showed highly similar absolute numbers for the number of viable cells in each sample, when eight different PBMC donors were tested (Supplemental Table 1).

Having said that, staining cells with Po-Pro-1™ involves complex mechanisms since, cell membranes in different stages of viability differ in their relative permeability to the three dyes. Intact cell membranes of live cells are permeable to AO. Partially compromised membranes are permeable to Po-Pro-1™, whereas only completely compromised cell membranes are permeable to PI. Based on these characteristics PoPro-1 exhibits overlap in its staining of some dead cells as well. Cells that stain with AO and Po-Pro-1™ are apoptotic, and cells that stain with Po-Pro-1™ and PI are dead. Cells that stain with AO and PI are also dead, as are cells that are stained with all three dyes. This staining pattern was de-convoluted by software developed by CTL for automated live, dead and apoptotic cell analysis using these three dyes. For the detection of apoptotic cells, images are taken with the above setting, and superimposed to detect cells that are stained with each of the three dyes. Based on the above staining patterns, live, dead, and apoptotic cell populations are defined. This analysis process was automated for high throughput counting as the Live/Dead/Apoptotic (LDA) cell counting software suite for ImmunoSpot® Analyzers.

### 3.2. Inducing Apoptosis in B Cells

We induced apoptosis in B cells to verify the accuracy of LDA counting, while also taking preparatory steps towards studying the impact of apoptotic bystander cells on T cell activation. B cells in PBMC can be targeted using the anti-CD20 antibody, Rituximab [29,30] that induces apoptosis in these cells [29,31,32]. We thawed cryopreserved PBMC and isolated B cells from these PBMC using anti-CD20-coupled magnetic dynabeads. The B cell depleted PBMC fraction and the isolated B cells were incubated overnight at 37 °C in the presence of Rituximab. Prior to, and following the overnight incubation, the numbers of live, dead, and apoptotic cells were determined using the LDA platform.

**Figure 2:**
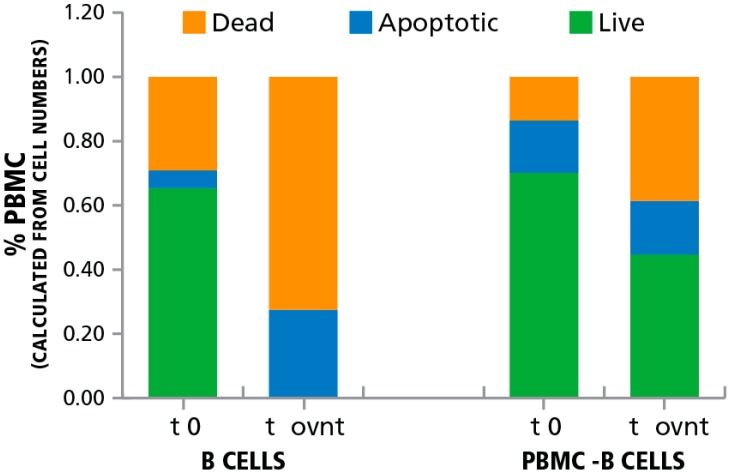
Induction of apoptosis in B cells using anti-CD20 coupled magnetic beads. B cells were isolated from PBMC using anti-CD20 coupled magnetic beads and were incubated overnight. The numbers of live, dead, and apoptotic cells was established right after separation (t 0) and after an overnight culture with anti-CD20 antibody (t ovnt). The percentage of live, dead, and apoptotic cells as detected by LDA staining are shown for the B cells and the PBMC population depleted of B cells (PBMC-B cells).

As seen in Figure 2, in the B cell depleted PBMC fraction of the thawed cells 70% of the cells were live, 14% dead, and were 16% apoptotic before storage. After the overnight storage 45% were live 39% were dead, and 16% were apoptotic. In the purified B cells, in contrast, after the overnight incubation, all the cells were either dead (73%) or dying (27% apoptotic). The data show that, where ongoing, apoptosis can be detected. In addition, the data shows that Rituximab treatment is suited for inducing apoptosis in B cells. Therefore, using this approach we were able to selectively induce apoptosis in B cells contained within PBMC without affecting the viability of the T cell population.

### 3.3. Apoptotic Bystander B Cells Do Not Affect T Cell Function

Multiple studies in the past have elucidated the mechanism of apoptosis and have studied the effect of apoptotic cells on various microenvironments [18,33]. However, there has been no study thus far that investigates T cell function in the presence of bystander apoptotic cells. As described in the Introduction, apoptotic cells activate macrophages that in turn secrete immune-suppressive cytokines. In the setting of a T cell assay, both could be expected to affect T cell function.

#### 3.3.1. Lack of Paracrine Effects

**Figure 3:**
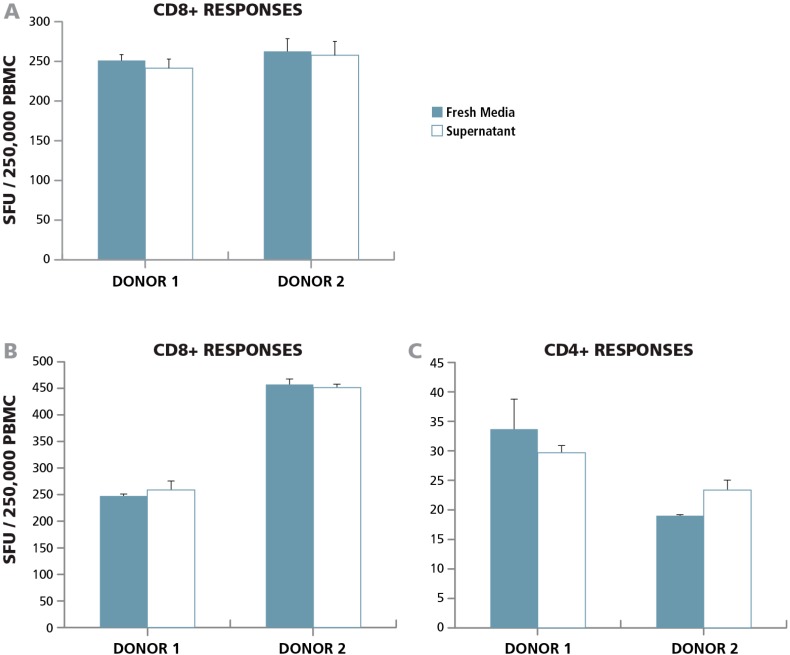
Bystander apoptotic cells do not exert a paracrine effect on T cell activation. (**A**) B cells isolated from PBMC were incubated overnight with monoclonal anti-CD20 antibody (Rituximab) and the supernatant was added to an IFN-γ ELISPOT assay in which PBMC were stimulated with the CEF peptide pool. Spot formation in the presence of fresh culture medium (solid bars), or with the apoptotic supernatant (open bars) was compared for the two PBMC donors specified. (**B** and **C**) Two PBMC donors were rested overnight at 37 °C, and the supernatant form the PBMC samples was added to an IFN-γ ELISPOT assay to detect recall responses from (**B**) CD8 cells (using CEF peptide pool antigen) or (**C**) CD4 cells (using Mumps antigen). The number of spot forming units in the presence of fresh medium (solid bars) or supernatant from rested cells (open bars) was analyzed for the two donors.

To study whether apoptotic B cells exert a paracrine effect on T cell function, we isolated B cells from PBMC and incubated them with Rituximab overnight. We centrifuged the cells and collected the supernatant that contained the signaling molecules secreted by the apoptotic B cells. Untreated PBMC were incubated with the apoptotic B cell supernatant in a 24 h human IFN-γ ELISPOT assay, and the CD8 cell response to CEF peptide pool was assessed. Surprisingly, we did not observe any change in T cell function compared to the control which was incubated with fresh medium (Figure 3A) demonstrating the absence of a detectable paracrine effect by apoptotic B cells on T cell function.

To further investigate the potential paracrine effects of apoptotic cells, PBMC were kept in a 37 °C incubator overnight. Such “overnight resting” leads to ~ 50% loss in PBMC [34], which is also visible in Figure 2 for the B cell-depleted PBMC. This loss of cells, due to apoptosis, will result in the engulfment of apoptotic cells by macrophages leading to the release of anti-inflammatory cytokines. Following overnight resting, the supernatant was collected. Untreated PBMC were incubated in media alone, or with the supernatant of apoptotic cells in a 24 h human IFN-γ ELISPOT assay measuring the recall response elicited by the CEF peptide pool ( which activates CD8 cells) and Mumps antigen (which activates CD4 cells). Again, we did not observe any change in CD4 or CD8 T cell function compared to control well treated with fresh medium (Figure 3B,C). In a related study by Lenders et all, apoptotic cells were not found to have an effect on the CD8 cell recall response to CMVpp65, but an effect was noted on the detection of CD4 cell responses to viral antigens, VZV or CMV cell lysates, that required antigen processing (34). We did not see an effect on mumps antigen-specific CD4 cells that also require antigen processing and presentation.

Both of these observations suggest that the presence of apoptotic bystander cells does not exhibit a paracrine effect on T cell functionality.

#### 3.3.2. Lack of Cell Contact-Mediated Effect

**Figure 4:**
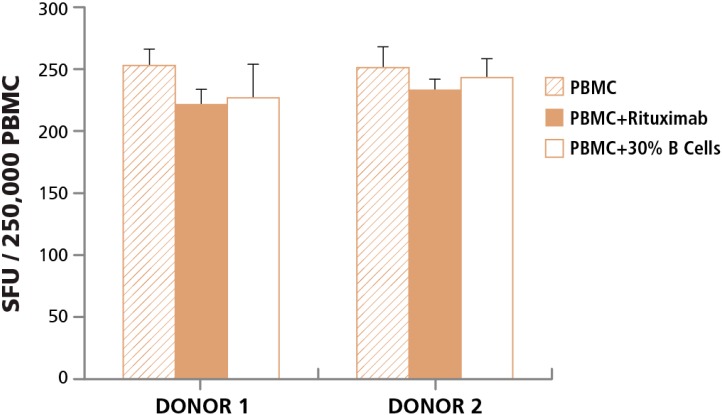
Cell-to-Cell contact of T cells with apoptotic B cells does not affect T cell activation. The specified two PBMC donors were tested in a human IFN-γ ELISPOT assay with CEF peptide pool added to activate CD8 cells (hatched bars). The PBMC were tested in parallel with anti-CD20 monoclonal antibody added to the wells during the ELISPOT assay (closed bars). To increase the number of apoptotic cells, B cells from the respective donors were isolated and 30% apoptotic B cells were added, while keeping the number of PBMC plated the same as above (open bars).

In order to understand whether cell-to-cell contact with apoptotic by-stander cells affects T cell functionality, we tested healthy T cells in the presence of B cells undergoing apoptosis. A 24 h human IFN-γ ELISPOT assay was performed, with 2.5 × 10^5^ PBMC per well, and CEF peptide pool added to elicit a CD8 cell response with and without adding Rituximab to the assay. Since in a typical PBMC sample B cells comprise only 5%–10% of the cell population, we also isolated B cells from the same PBMC donor and added 30% more B cells (while keeping the number of PBMC added to each experimental condition the same) thereby increasing the numbers of cells actively undergoing apoptosis when the same number of T cells gets activated. We found no significant change in T cell functionality when T cells came in contact with excessive numbers of apoptotic cells (Figure 4).

Bystander apoptotic cells, therefore, do not seem to affect T cell function in ELISPOT assays. The data suggest that eliminating apoptotic cells from PBMC will not improve T cell assay results. However, measuring the numbers of apoptotic cells in PBMC gives an overall indication of the damage that PBMC, including the T cells contained in the PBMC, have suffered during PBMC processing. The data also imply that if T cell reactivity is reduced in PBMC samples that contain a high number of apoptotic cells, it is most likely due to damage to T cells themselves rather than an effect exerted by bystander cells.

### 3.4. Post- Rather than Pre-T cell Assay Detection of Apoptotic Cells in PBMC Indicates Impaired T Cell Function

**Figure 5:**
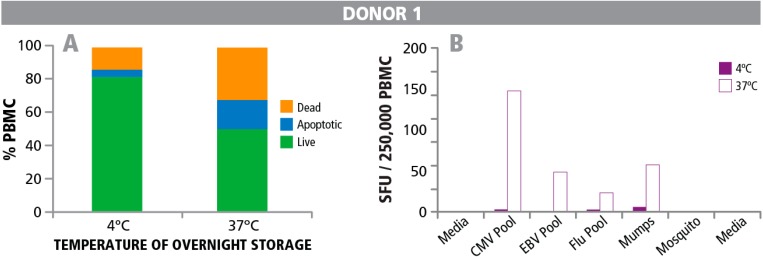
Percentage of live, dead, and apoptotic T cells, and T cell functionality after storing PBMC at 4 °C or 37 °C overnight. PBMC were stored at 4 °C or 37 °C overnight. (**A**) The numbers of live, dead, and apoptotic cells was determined after overnight storage. (**B**) PBMC stored overnight at 4 °C (solid bars) and 37 °C (open bars) were resuspended in fresh medium and tested in an IFN-γ ELISPOT assay with different antigens. CMV peptide pool, EBV peptide pool, and Flu peptide pool were used to activate CD8 cells and Mumps and Mosquito antigens to activate CD4 cells.

From previous observations we were aware that PBMC show decreased functionality when stored at 4 °C overnight *vs.* at 37 °C [9,14,35]. We decided to take a closer look at this empirical finding. Cryopreserved PBMC were thawed under optimal conditions, and split in two aliquots that were stored at either 37 °C or 4 °C overnight. The next day, cells were counted to determine the numbers of live, dead, and apoptotic cells before plating them into an ELISPOT assay. For the cells stored at 4 °C, the percentage of live cells was substantially higher than for those stored at 37 °C. Data are shown for one representative PBMC donor in Figure 5A. The percentages of live cells at the two temperatures for this donor were 82% *vs*. 50%, respectively. Data for all 9 PBMC donors tested can be seen in Supplementary Figure 1. The number of dead and apoptotic cells was substantially higher for the cells stored at 37 °C (Figure 5A, and Supplementary Figure 1). Also, cell recovery was higher at 4 °C. For the donor shown in Figure 5A, the recovery was 9.76 × 10^6^ viable cells when stored at 4 °C *vs.* 7.22 × 10^6^ cells when stored at 37 °C. Recovery rates for the other donors under the two storage conditions can be found in Supplementary Table 2. If the measurement of cell recovery, viability and frequency of apoptotic cells in PBMC done prior to testing T cells would be predictive of T cell functionality, PBMC stored at 4 °C should reveal the higher response levels.

However, we found the opposite observation when measuring T cell functional responses. T cell functionality was greatly reduced in the samples stored at 4 °C compared to those that were stored at 37 °C (Figure 5A,B). Both CD8 responses (recalled by CMV peptide pool, EBV peptide pool, and Flu peptide pool) and CD4 responses (recalled by Mumps and Mosquito antigen) were essentially lost in the PBMC stored at 4 °C. This observation was made in all nine out of the nine PBMC donors tested (see Supplemental Figure 1). Following the general acceptance criteria [14] nine out of nine sample at 4 °C with greater than 89% viability and minimal apoptotic cells would have passed while all nine of nine samples stored at 37 °C with viability less than 89% would have failed.

**Figure 6:**
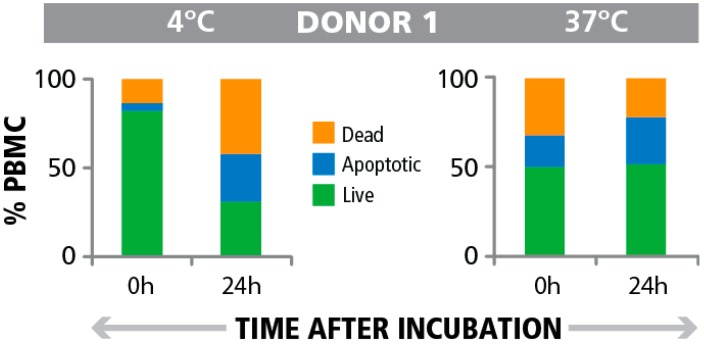
Frequency of apoptotic cells pre- and post PBMC testing in the ELISPOT assay. PBMC were stored at 4 °C or 37 °C overnight. The numbers of live, dead, and apoptotic cells (and their respective percentages show in the specified colors) was recorded after overnight storage, prior to and at the end of a 24 h IFN-γ ELISPOT assay.

Attempting to resolve this apparent contradiction, we tested the hypothesis that consequences of PBMC damage might manifest only over time. We therefore determined the numbers of live, dead, and apoptotic cells in the PBMC samples over the course of the incubation period of the T cell assay. As shown in Figure 6 for a representative donor (and in the Supplemental Figure 2 for the additional eight donors) that had been tested, the samples stored at 4 °C deteriorated over the 24 h time period and in some cases there were no live cells detectable after 24 h. In contrast, the samples stored at 37 °C maintained their viability over the 24 h time period of the T cell assay with minimal change. For the representative donor shown here, the number (%) of apoptotic cells in the samples stored at both 4 °C and 37 °C, at time 24 h, was similar. However the number of dead cells was greatly increased for the samples stored at 4 °C. The likely explanation for this finding is that apoptosis is a transient process leading to cell death. Therefore when observed at a single time point, only those cells that are actively undergoing apoptosis at that time will be detected as apoptotic cells and those that underwent apoptosis earlier will appear in the dead cell category. This finding shows that the actual damage to the samples and the subsequent loss of T cell function can become evident only with time, and can be detected through measurements over time, and not simply at onset of the assay.

During sample processing, shipment, and cryopreservation individual samples may be handled differently and may suffer damages even when similar protocols are followed. Even simple processing steps, such as bringing a cryopreserved sample to 37 °C during the thawing process and the time for thawing, can greatly affect sample functionality. In other words, one cannot predict whether a sample will be functional prior to the start of an assay merely by detecting viability. Under these conditions, post assay apoptotic measurements will reveal whether the sample was damaged and therefore, whether the functional data are valid.

Overnight resting of samples can occasionally help correct for the increased apoptotic rate in sub-optimal samples. However, resting rarely leads to more than two fold increase in ELISPOT reactivity, relative to unrested sample. Moreover, as shown in this study, there is no active bystander effect of apoptotic cells. Since approximately half of the cells are lost in overnight resting, while cell numbers plated and antigen-induced spot counts are linearly related (and the background does not increase), with plating more cells it seems more prudent to just plate more cells instead of resting.

For 24 h ELISPOT assays, pre- and post-assessment of viability and apoptosis numbers are recommended at 0 h and 24 h for quality control of PBMC function. It will need to be established whether extending this observation period beyond 24 h further benefits quality control for this functional assay and whether such extensions would be required for assays for longer durations such as proliferation, killing, or establishing cell lines.

## 4. Conclusions

Based on the data presented here, we suggest that serial measurements of live, dead, and apoptotic cell numbers in PBMC samples provide more accurate acceptance criteria regarding the quality PBMC samples, as opposed to a single measurement of these parameters prior to performing a T cell assay. As the numbers of PBMC available for testing is frequently limiting in clinical trials, an attractive possibility for the second live/dead apoptotic cell count is to test the PBMC that were plated in an ELISPOT assay following the cell incubation period. PBMC survive the ELISPOT assay unharmed, just as they would when plated separately into 96 well cell culture plates. Typically, these PBMC are discarded after the incubation period, before adding the reagents required for detecting the plate-bound analyte. Instead of discarding them, we suggest assessing the numbers of live, dead, and apoptotic cells in each well, which can be done readily and rapidly with CTL’s LDA cell counting platform. Our data also show that apoptosis of bystander cells does not affect T cell function. Neither contact with apoptotic APC, nor signaling molecules secreted by apoptotic cells or macrophages seem to have an effect on T cell function. This finding suggests that there might be no benefit to removing apoptotic cells from PBMC samples.

## Supplementary Materials

Supplementary materials can be accessed at: http://www.mdpi.com/2073-4409/4/1/40/s1.

## References

[1] Kreher C.R., Dittrich M.T., Guerkov R., Boehm B.O., Tary-Lehmann M. (2003). CD4+ and CD8+ cells in cryopreserved human pbmc maintain full functionality in cytokine ELISPOT assays. J. Immunol. Methods.

[2] Axelsson S., Faresjo M., Hedman M., Ludvigsson J., Casas R. (2008). Cryopreserved peripheral blood mononuclear cells are suitable for the assessment of immunological markers in type 1 diabetic children. Cryobiology.

[3] Maecker H.T., Moon J., Bhatia S., Ghanekar S.A., Maino V.C., Payne J.K., Kuus-Reichel K., Chang J.C., Summers A., Clay T.M. (2005). Impact of cryopreservation on tetramer, cytokine flow cytometry, and ELISPOT. BMC Immunol..

[4] Weinberg A., Song L.Y., Wilkening C.L., Fenton T., Hural J., Louzao R., Ferrari G., Etter P.E., Berrong M., Canniff J.D. (2010). Optimization of storage and shipment of cryopreserved peripheral blood mononuclear cells from HIV-infected and uninfected individuals for ELISPOT assays. J. Immunol. Methods.

[5] Peters B.S., Jaoko W., Vardas E., Panayotakopoulos G., Fast P., Schmidt C., Gilmour J., Bogoshi M., Omosa-Manyonyi G., Dally L. (2007). Studies of a prophylactic HIV-1 vaccine candidate based on modified vaccinia virus ankara (MVA) with and without DNA priming: Effects of dosage and route on safety and immunogenicity. Vaccine.

[6] Garcia S., Lagos R., Munoz A., Picon T., Rosa R., Alfonso A., Abriata G., Gentile A., Romanin V., Regueira M. (2012). Impact of vaccination against haemophilus influenzae type B with and without a booster dose on meningitis in four south american countries. Vaccine.

[7] Basha S., Hazenfeld S., Brady R.C., Subbramanian R.A. (2011). Comparison of antibody and T-cell responses elicited by licensed inactivated- and live-attenuated influenza vaccines against H3N2 hemagglutinin. Hum. Immunol..

[8] Yang F.F., Huang W., Li Y.F., Gao Z.G. (2011). Current status of non-viral vectors for sirna delivery. Yao Xue Xue Bao.

[9] Ramachandran H., Laux J., Moldovan I., Caspell R., Lehmann P.V., Subbramanian R.A. (2012). Optimal thawing of cryopreserved peripheral blood mononuclear cells for use in high-throughput human immune monitoring studies. Cells.

[10] Areman E.M., Simonis T.B., Carter C.S., Read E.J., Klein H.G. (1988). Bulk cryopreservation of lymphocytes in glycerol. Transfusion.

[11] Cox J.H., Ferrari G., Janetzki S. (2006). Measurement of cytokine release at the single cell level using the ELISPOT assay. Methods.

[12] Gill D.K., Huang Y., Levine G.L., Sambor A., Carter D.K., Sato A., Kopycinski J., Hayes P., Hahn B., Birungi J. (2010). Equivalence of ELISPOT assays demonstrated between major HIV network laboratories. PloS One.

[13] Zhang W., Caspell R., Karulin A.Y., Ahmad M., Haicheur N., Abdelsalam A., Johannesen K., Vignard V., Dudzik P., Georgakopoulou K. (2009). ELISPOT assays provide reproducible results among different laboratories for T-cell immune monitoring—even in hands of ELISPOT-inexperienced investigators. J. Immunotoxicol..

[14] Smith J.G., Joseph H.R., Green T., Field J.A., Wooters M., Kaufhold R.M., Antonello J., Caulfield M.J. (2007). Establishing acceptance criteria for cell-mediated-immunity assays using frozen peripheral blood mononuclear cells stored under optimal and suboptimal conditions. Clin. Vaccine Immunol..

[15] Mascotti K., McCullough J., Burger S.R. (2000). Hpc viability measurement: Trypan blue *versus* acridine orange and propidium iodide. Transfusion.

[16] Mackenzie A.B., Young M.T., Adinolfi E., Surprenant A. (2005). Pseudoapoptosis induced by brief activation of atp-gated P2X7 receptors. J. Biol. Chem..

[17] Cankurtaran-Sayar S., Sayar K., Ugur M. (2009). P2x7 receptor activates multiple selective dye-permeation pathways in raw 264.7 and human embryonic kidney 293 cells. Mol. Pharmacol..

[18] Savill J., Dransfield I., Gregory C., Haslett C. (2002). A blast from the past: Clearance of apoptotic cells regulates immune responses. Nat. Rev. Immunol..

[19] Fadok V.A., Voelker D.R., Campbell P.A., Cohen J.J., Bratton D.L., Henson P.M. (1992). Exposure of phosphatidylserine on the surface of apoptotic lymphocytes triggers specific recognition and removal by macrophages. J. Immunol..

[20] Torr E.E., Gardner D.H., Thomas L., Goodall D.M., Bielemeier A., Willetts R., Griffiths H.R., Marshall L.J., Devitt A. (2012). Apoptotic cell-derived ICAM-3 promotes both macrophage chemoattraction to and tethering of apoptotic cells. Cell Death Differ..

[21] Savill J.S., Henson P.M., Haslett C. (1989). Phagocytosis of aged human neutrophils by macrophages is mediated by a novel “harge-sensitive” ecognition mechanism. J. Clin. Invest..

[22] Lauber K., Bohn E., Krober S.M., Xiao Y.J., Blumenthal S.G., Lindemann R.K., Marini P., Wiedig C., Zobywalski A., Baksh S. (2003). Apoptotic cells induce migration of phagocytes via caspase-3-mediated release of a lipid attraction signal. Cell.

[23] Scannell M., Flanagan M.B., deStefani A., Wynne K.J., Cagney G., Godson C., Maderna P. (2007). Annexin-1 and peptide derivatives are released by apoptotic cells and stimulate phagocytosis of apoptotic neutrophils by macrophages. J. Immunol..

[24] Truman L.A., Ford C.A., Pasikowska M., Pound J.D., Wilkinson S.J., Dumitriu I.E., Melville L., Melrose L.A., Ogden C.A., Nibbs R. (2008). Cx3cl1/fractalkine is released from apoptotic lymphocytes to stimulate macrophage chemotaxis. Blood.

[25] Bournazou I., Pound J.D., Duffin R., Bournazos S., Melville L.A., Brown S.B., Rossi A.G., Gregory C.D. (2009). Apoptotic human cells inhibit migration of granulocytes via release of lactoferrin. J. Clin. Invest..

[26] Voll R.E., Herrmann M., Roth E.A., Stach C., Kalden J.R., Girkontaite I. (1997). Immunosuppressive effects of apoptotic cells. Nature.

[27] Fadok V.A., Bratton D.L., Konowal A., Freed P.W., Westcott J.Y., Henson P.M. (1998). Macrophages that have ingested apoptotic cells *in vitro* inhibit proinflammatory cytokine production through autocrine/paracrine mechanisms involving TGF-beta, PGE2, and PAF. J. Clin. Invest..

[28] Lehmann P.V. (2005). Image analysis and data management of ELISPOT assay results. Methods Mol. Biol..

[29] Shan D., Ledbetter J.A., Press O.W. (1998). Apoptosis of malignant human B cells by ligation of Cd20 with monoclonal antibodies. Blood.

[30] Pedersen I.M., Buhl A.M., Klausen P., Geisler C.H., Jurlander J. (2002). The chimeric anti-CD20 antibody rituximab induces apoptosis in B-cell chronic lymphocytic leukemia cells through a p38 mitogen activated protein-kinase-dependent mechanism. Blood.

[31] Collins D.M., O’Donovan N., McGowan P.M., O’Sullivan F., Duffy M.J., Crown J. (2012). Trastuzumab induces antibody-dependent cell-mediated cytotoxicity (ADCC) in her-2-non-amplified breast cancer cell lines. Ann. Oncol. ESMO.

[32] Kimura H., Sakai K., Arao T., Shimoyama T., Tamura T., Nishio K. (2007). Antibody-dependent cellular cytotoxicity of cetuximab against tumor cells with wild-type or mutant epidermal growth factor receptor. Cancer Sci..

[33] Hochreiter-Hufford A., Ravichandran K.S. (2013). Clearing the dead: Apoptotic cell sensing, recognition, engulfment, and digestion. Cold Spring Harbor Perspect. Biol..

[34] Kuerten S., Batoulis H., Recks M.S., Karacsony E., Zhang W., Subbramanian R.A., Lehmann P.V. (2012). Resting of cryopreserved PBMC does not generally benefit the performance of antigen-specific T cell ELISPOT assays. Cells.

[35] Lenders K., Ogunjimi B., Beutels P., Hens N., Van Damme P., Berneman Z.N., Van Tendeloo V.F., Smits E.L. (2010). The effect of apoptotic cells on virus-specific immune responses detected using IFN-gamma ELISPOT. J. Immunol. Methods.

