# Supplementary material for: Serial Measurements of Apoptotic Cell Numbers Provide Better Acceptance Criterion for PBMC Quality than a Single Measurement Prior to the T Cell Assay
Source: Cells. 2015 Jan 9;4(1):40–55. doi: 10.3390/cells4010040 (PMC4381208; doi:10.3390/cells4010040)
Supplement: Supplementary file 1 [file cells-04-00040-s001.pdf]

Supplementary Material

Table S1. Comparison of Viability of PBMC between Manual Eye Counts and CTL Analyzer Counts. Eight PBMC samples were stained with AO and PI. They were counted by eye under a microscope, and counted with the Analyzer using the L/D/A software.

	**Manual Visual Count**	**CTL Automated Analyzer Count**
Donor 1	1.40×10^7	1.49×10^7
Donor 2	1.63×10^7	1.67×10^7
Donor 3	1.69×10^7	1.61×10^7
Donor 4	1.59×10^7	1.50×10^7
Donor 5	1.37×10^7	1.30×10^7
Donor 6	1.41×10^7	1.40×10^7
Donor 7	1.36×10^7	1.42×10^7
Donor 8	1.45×10^7	1.38×10^7

Table S2. Recovery of PBMC after overnight resting. Nine cryopreserved PBMC samples were thawed and rested overnight at 4 °C and 37 °C. PBMC were stained with AO, PI, and Po-Pro-1™ dyes and the number of viable cells in the samples was computed using the L/D/A software.

**Cell Recovery After Resting Overnight**		
	4 °C	37 °C
Donor 1	9.76×10^6	7.22×10^6
Donor 2	9.32×10^6	3.62×10^6
Donor 3	9.75×10^6	8.34×10^6
Donor 4	1.36×10^7	1.12×10^7
Donor 5	9.14×10^6	6.47×10^6
Donor 6	1.25×10^7	1.21×10^7
Donor 7	1.61×10^7	1.05×10^7
Donor 8	2.33×10^7	2.09×10^7
Donor 9	1.60×10^7	1.13×10^7

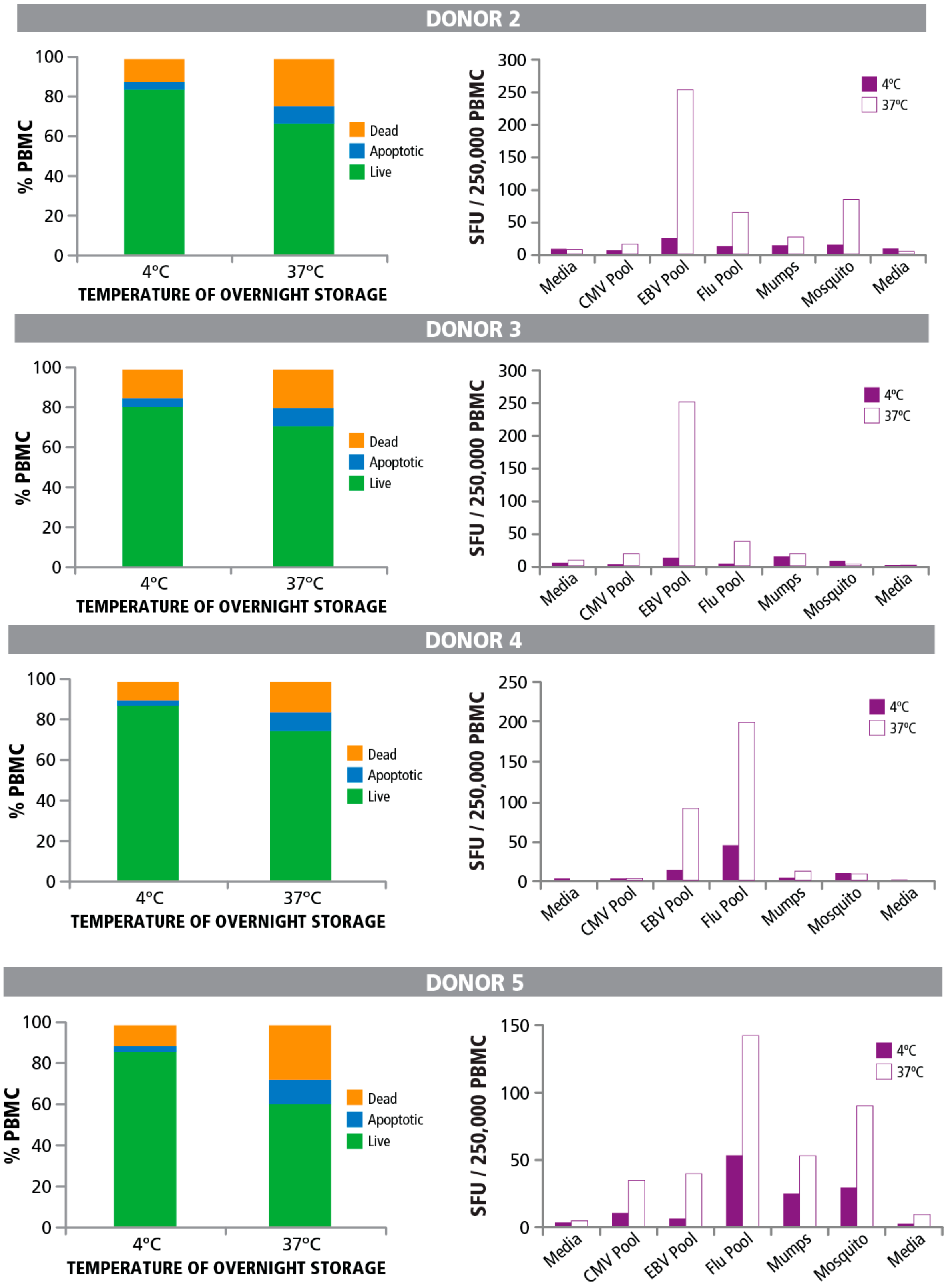

Figure S1. *Cont.*

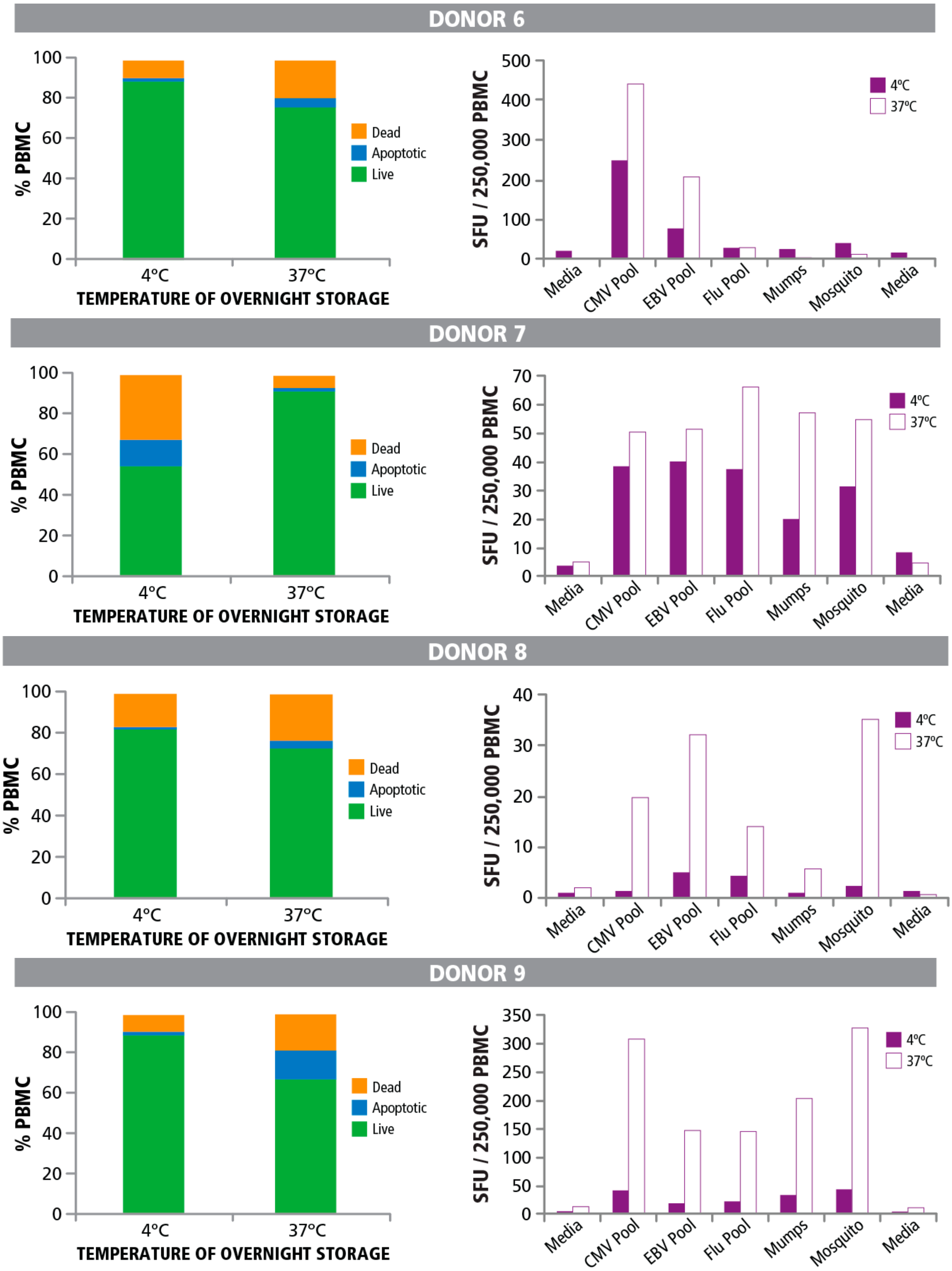

Figure S1. PBMC were stored at 4 °C or 37 °C overnight. Data from Donors 2–9 are shown here (**A**) The numbers of live, dead, and apoptotic cells was determined after overnight storage. (**B**) PBMC stored overnight at 4 °C (solid bars) and 37 °C (open bars) were resuspended in fresh medium and tested in an IFN-γ ELISPOT assay with different antigens. CMV peptide pool, EBV peptide pool, and Flu peptide pool were used to activate CD8 cells and Mumps and Mosquito antigens to activate CD4 cells.

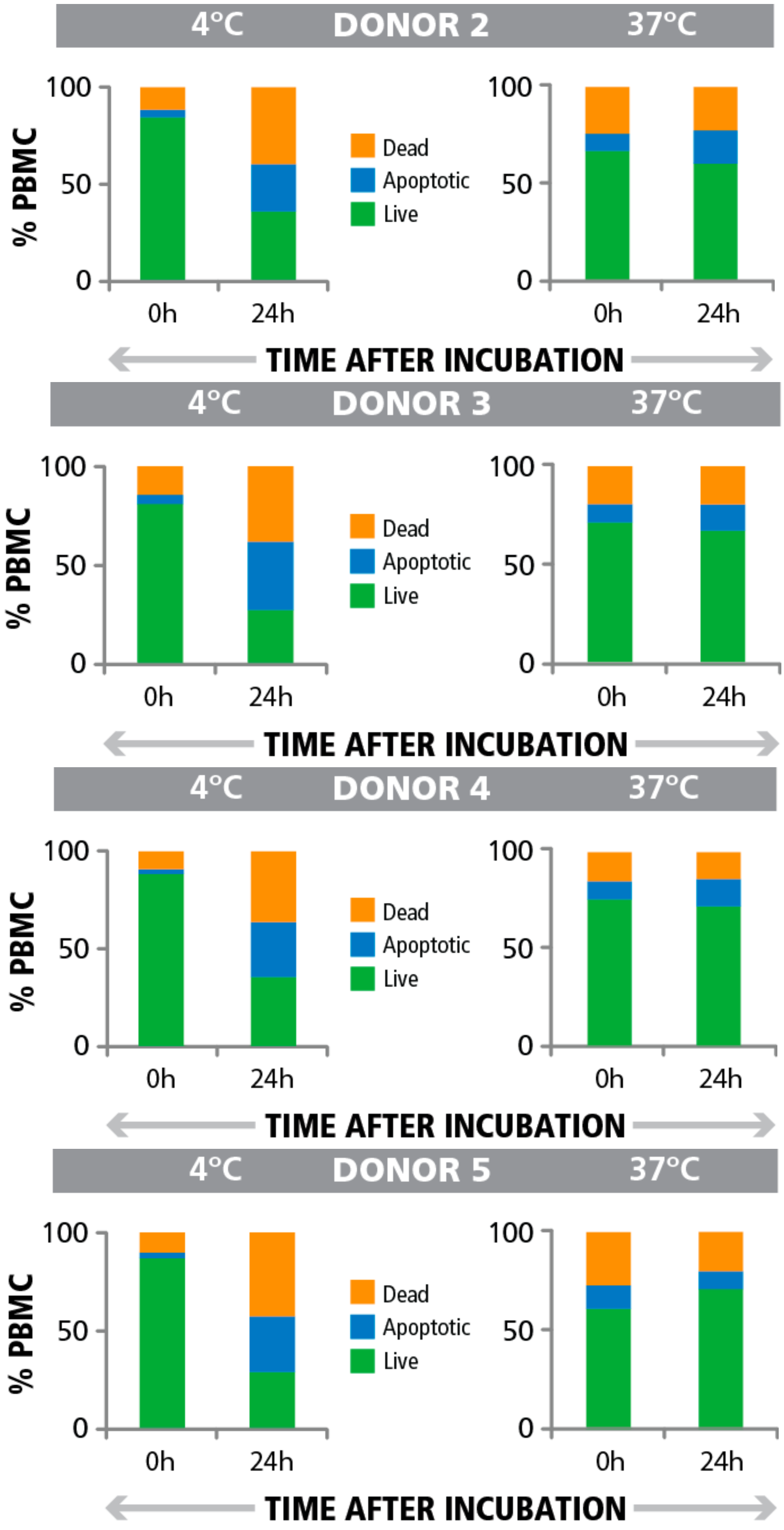

Figure S2. *Cont.*

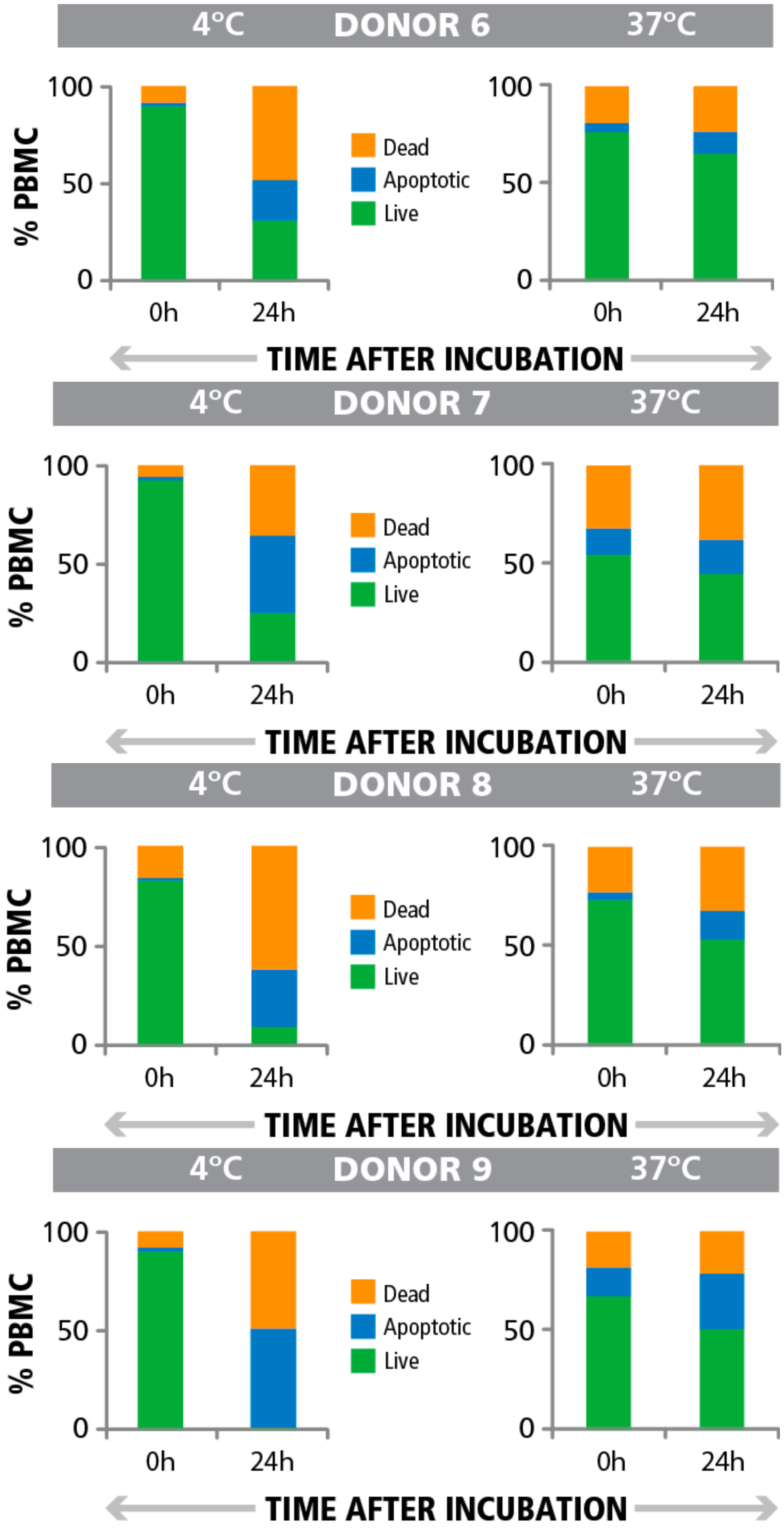

Figure S2. PBMC were stored at 4 °C or 37 °C overnight. Data from Donors 2–9 are shown here. The numbers of live, dead, and apoptotic cells (and their respective percentages show in the specified colors) was recorded after overnight storage, prior to and at the end of a 24 h IFN-γ ELISPOT assay.
